# Integrated multi-omics analysis identifies SELENOP and PKMYT1 as immune-metabolic hub genes in breast cancer

**DOI:** 10.1016/j.bbrep.2025.102198

**Published:** 2025-08-06

**Authors:** Guohui Tang, Zheng Zhang, Bo Pang, Ruonan Li, Yuting Liu, Haotian Cai, Wenrui Wang, Changjie Chen, Yurong Ou, Qingling Yang

**Affiliations:** a Anhui Provincial Key Laboratory of Tumor Evolution and Intelligent Diagnosis and Treatment (Bengbu Medical University), Anhui, 233030, China; b Department of Life Sciences, Bengbu Medical University, Anhui, 233030, China; c Department of Pathology, The First Affiliated Hospital of Bengbu Medical University, Anhui, 233030, China; d Department of Biochemistry and Molecular Biology, Bengbu Medical University, Anhui, 233030, China; e Institute of Health and Medicine, Hefei Comprehensive National Science Center, Hefei, Anhui, China

**Keywords:** Breast cancer, WGCNA, Biomarkers, Machine learning

## Abstract

**Background:**

Metabolic reprogramming and immune evasion synergistically drive breast carcinogenesis, but their combined impact remains unclear.

**Methods:**

Transcriptomic data from the TCGA and GEO cohorts were integrated. Differentially expressed genes were identified, followed by WGCNA to detect immune-correlated co-expression modules. Immune-metabolism-related genes (IMGs) were screened using Genecards. Four machine learning algorithms (LASSO, SVM, RF, XGBoost) identified hub genes. The diagnostic value was evaluated by Kaplan-Meier and ROC analysis. CIBERSORT quantified immune microenvironment associations. The expression profiles of genes in different cells were plotted using single-cell RNA data. IHC validated protein expression in clinical samples.

**Results:**

Research has found that SELENOP and PKMYT1 are key immune metabolic hubs. Compared with normal tissues, the expression of SELENOP was significantly decreased (p < 0.05), while PKMYT1 showed an upward trend (p < 0.05). Both of these genes have demonstrated high accuracy in the diagnosis of breast cancer and can effectively predict the overall survival period of patients. Low SELENOP expression is associated with high PKMYT1 expression levels, which is significantly related to changes in immune infiltration and the expression patterns of checkpoint proteins. Immunohistochemical detection further confirmed that these genes were significantly correlated with histological grade, LAG-3, CD244, ER, PR and Her-2 and other indicators (p < 0.05).

**Conclusion:**

SELENOP and PKMYT1 are novel immunomodulatory factors related to multiple pathological indicators of breast cancer and can be used as diagnostic biomarkers.

## Highlights

• SELENOP/PKMYT1 was identified as an immune-metabolic center gene through comprehensive bioinformatics.
• The expression and survival impact of key genes were verified using dual datasets.
• The IHC of clinical samples confirmed the diagnostic and prognostic value.

## 1 Introduction

Breast cancer (BC) is the most commonly diagnosed malignancy in women [1], with immune evasion and metabolic reprogramming being two major characteristics of the disease [2]. While the immune system eliminates transformed cells through multiple effector mechanisms (including cytotoxicity, phagocytosis, and cytokine-mediated surveillance) [3], BC cells disrupt immune surveillance by overexpressing immunosuppressive ligands such as PD-L1. This process induces apoptosis and functional exhaustion of tumor-specific CD8^+^ T cells, thereby ultimately leading to their impairment [4]. Emerging evidence suggests that there is complex crosstalk between immune dysfunction and metabolic dysregulation in BC progression [5]. As tumors develop, cancer cells reprogram their metabolism in response to the nutrient-poor tumor microenvironment, utilizing metabolic reprogramming through glycolysis, oxidative phosphorylation, amino acid metabolism, lipid metabolism, and other metabolic pathways to meet bioenergetic and biosynthetic demands [6,7].

**Fig. 1:**
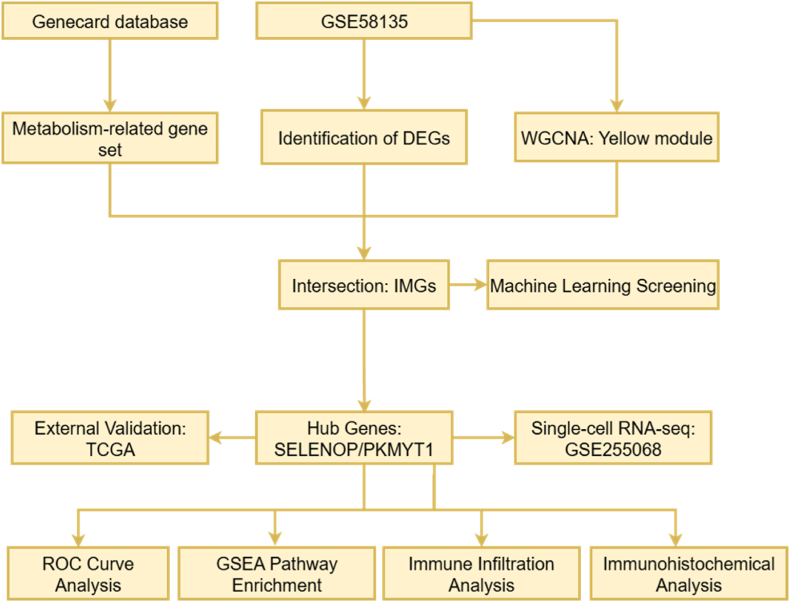
Research flowchart.

Given the core role of immune evasion and metabolic reprogramming in the progression of breast cancer and their complex interactions, understanding how metabolic reprogramming promotes cancer progression by influencing the immune system is of significant importance in unraveling the complex mechanisms of cancer. To identify the key molecules mediating the interaction between immune evasion and metabolic reprogramming in breast cancer, this study utilized TCGA and GEO data, applying WGCNA and machine learning algorithms to screen out SELENOP and PKMYT1 genes closely related to immunity and metabolism, as shown in Fig. 1. SELENOP, primarily a selenium transporter and antioxidant [8], is implicated in modulating inflammatory responses and immune cell activity within the tumor microenvironment, linking selenium/redox metabolism to immune regulation [9,10]. PKMYT1, a critical cell cycle regulator at the G2/M checkpoint [11], has emerging but less defined roles beyond proliferation, with potential intersections in metabolic signaling and tumor microenvironment influence. However, their specific contributions to the interplay between immune evasion and metabolic reprogramming in breast cancer, and the resulting prognostic significance, remain poorly understood.

## 2 Materials and methods

### 2.1 Cancer data sources

The GSE58135 and GSE161529 datasets were downloaded from GEO (https://www.ncbi.nlm.nih.gov/geo/). GSE58135 (GPL11154 platform) contains 168 breast tissue samples. The scRNA-seq data under accession number GSE161529 was selected, including 28 samples: 4 from TNBC, 6 from HER + tumors, and 18 from ER + tumors. Additionally, 23,931 metabolism-related genes were downloaded from the Genecardss database.

### 2.2 Identification of DEGs

DEG analysis was performed on GSE58135 using the “limma” R package with thresholds of |log2 fold change| > 3 and false discovery rate (FDR) < 0.01, resulting in 1026 significant DEGs. Visualization was performed using the 'ggplot2' package, with a volcano plot generated to illustrate the results.

### 2.3 WGCNA screening for immune-related genes

Weighted Gene Co-Expression Network Analysis (WGCNA) is a systems biology approach used to identify co-expressed gene modules and explore the relationship between these modules and phenotypic traits [12]. This module-based approach efficiently identifies genes acting in concert with immune processes, avoiding limitations of single-gene analysis. First, we constructed a gene co-expression network for breast cancer tissue samples using the WGCNA R package, which was then divided into different modules. Next, we used the CIBERSORT tool to obtain the immune cell distribution for each sample. Finally, we calculated the correlation between the module eigengenes (ME) and phenotypic variables, selecting the module with the strongest correlation to immune scores.

### 2.4 Screening hub genes using four machine learning methods

In this study, four machine learning algorithms were used to identify immune-metabolic hub genes biomarkers for breast cancer, including LASSO, SVM, RF, and XGBoost. LASSO is a data mining technique that applies an absolute value penalty to regression coefficients, shrinking some coefficients to zero and eliminating less important features [13]. SVM is a supervised learning algorithm that identifies important disease-associated genes by analyzing support vectors and decision boundaries [14]. RF is an ensemble learning method that evaluates the importance of each gene in classification, helping to identify genes associated with the disease [15]. XGBoost is a gradient boosting-based ensemble learning method that selects important genes based on feature importance scores [16]. Compared to conventional differential expression analysis, these machine learning algorithms excel at identifying key biomarkers by capturing complex, non-linear interactions and synergistic effects among genes, rather than relying solely on individual gene expression changes. The overlapping genes identified by these four algorithms were considered as candidate gene biomarkers.

### 2.5 scRNA-seq data processing and cell type identification

First, 28 breast cancer samples were included, with a total of 186,113 cells. The criteria for cell inclusion were set to include genes expressed in 500 to 5000 genes per cell, and the maximum percentage of mitochondrial genes was set at 15 %. After filtering, 87,597 cells were retained. The data from all 28 samples were then log-normalized. The FindVariableFeatures function was used to identify the top 2000 highly variable genes. Subsequently, cells were clustered using the FindNeighbors and FindClusters functions, which resulted in 12 distinct clusters. Gene annotations for the clusters were gathered by reviewing the literature. The clusters were then categorized into different cell types based on these annotations. This single-cell method can precisely identify gene expression patterns within specific cell subpopulations, providing more specific resolution compared to conventional tissue analysis. Finally, the expression of hub genes across different tissues was analyzed to evaluate their relevance and potential as biomarkers for breast cancer.

### 2.6 ROC analysis and survival analysis

Differential analysis of SELENOP and PKMYT1 was performed in the GSE58135 dataset, and validation was carried out in the TCGA breast cancer dataset. Additionally, receiver operating characteristic (ROC) curve analysis demonstrated the diagnostic accuracy of SELENOP and PKMYT1 in distinguishing breast cancer tissues from normal tissues. Survival analysis for breast cancer patients was conducted using the Kaplan-Meier Plotter(http://kmplot.com/analysis/) [17]. The Kaplan-Meier curve was used to assess the overall survival based on the expression levels of SELENOP and PKMYT1, providing insight into their prognostic value in breast cancer.

### 2.7 Immune infiltration analysis

CIBERSORT, an algorithm that estimates the relative abundance of immune cell types by evaluating subsets of RNA transcripts, was used to analyze immune cell infiltration [18]. This method uses a predefined reference gene expression matrix to infer the abundance of immune cell populations. A total of 22 immune cell types were evaluated, and the proportion and differential changes of immune cells between the tumor and normal groups were thoroughly analyzed and visualized. This approach provides a standardized and comprehensive view of the complex immune landscape within breast cancer tissues. The correlation between SELENOP and PKMYT1 and the 22 immune cell types was calculated.

### 2.8 Enrichment analysis

Pathway enrichment analysis was performed using gene set enrichment analysis (GSEA) to identify biological processes associated with SELENOP and PKMYT1 expression [19]. The tumor samples were divided into high expression group and low expression group according to the median expression level. Genes were ranked by log2 fold change between groups, and GSEA was performed against GO biological process terms (MSigDB c5.go.v7.4) with 1000 permutations. All analyses utilized the clusterProfiler R package.

### 2.9 Tissue samples

A total of 60 paraffin-embedded tissue samples from breast cancer patients were included in this study (The First Affiliated Hospital of Bengbu Medical University). The study was approved by the Ethics Committee of Bengbu Medical University, and informed consent was obtained from all patients.

### 2.10 Immunohistochemistry (IHC)

Immunohistochemistry was performed using the standard procedure. Tissue sections were prepared and underwent antigen retrieval and hydrogen peroxide blocking steps. Primary antibodies were applied for 1 h at 37 °C, followed by PBS washing to remove unbound antibodies. Secondary antibodies were added and incubated for 30 min at 37 °C, followed by PBS washing. The sections were then stained with DAB (3 min), and the reaction was terminated after the color developed. Hematoxylin was used for counterstaining (1 min), followed by rinsing with tap water. The slides were dehydrated through successive ethanol solutions (75 %, 85 %, 95 %, and 100 %) for 3 min each. Neutral balsam was applied to mount the slides, and a coverslip was placed to avoid air bubbles.

All IHC slides were independently scored by two breast pathologists with >5 years of specialization experience, blinded to clinical data. Discordant cases (score difference ≥2 points) underwent consensus review using a multi-head microscope to finalize scores. The staining results were observed under a light microscope. Images were captured and analyzed. The staining intensity was scored as follows: no color (0 points), pale yellow (1 point), brown-yellow (2 points), and brown (3 points). The percentage of positive staining was scored as 0 (negative), 1 (1–25 %), 2 (26–50 %), 3 (51–75 %), and 4 (76–100 %). The total score was the product of the “staining intensity score” and the “positive staining rate score."

### 2.11 Statistical analysis

Data analysis was performed using R software (version 4.2.1). Pearson correlation analysis was conducted to explore the relationship between hub genes and clinical characteristics. A p-value <0.05 was considered statistically significant for all analyses.

## 3 Results

### 3.1 GenesSelection of immune- and metabolism-related genes

**Fig. 2:**
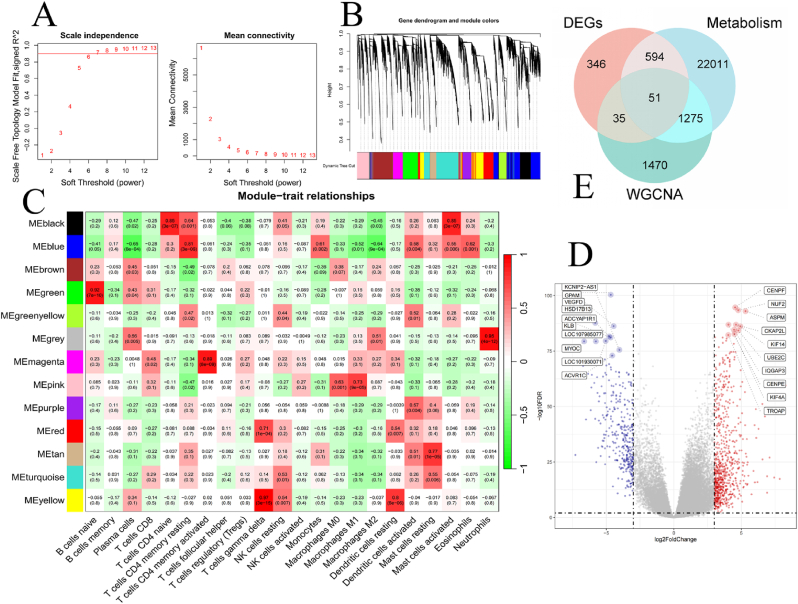
Acquisition of IMGs (A) Scatter plot of the scale-free topology fitting index and the mean connectivity plot for determining the optimal soft threshold. (B) Hierarchical clustering dendrogram based on the Topological Overlap Matrix (TOM), illustrating the gene modules. (C) Heatmap showing the correlation between gene modules and immune cell scores. (D) Volcano plot of differentially expressed genes, where 701 genes are upregulated and 325 genes are downregulated. (E) The Venn diagram illustrates the intersection of immune-related genes, metabolism-related genes, and genes screened in the “DESeq2″ R package, resulting in the identification of 51 immune-metabolic genes (IMGs).

To identify immune-related genes, we performed WGCNA on gene expression data from BRCA patients to construct a gene co-expression network. Initially, Pearson correlation coefficients were calculated for all genes to build the adjacency matrix and Topological Overlap Matrix (TOM). The optimal soft threshold for the scale-free topology model was determined to be 6 (Fig. 2A). A total of 39,376 genes were divided into 13 modules(Fig. 2B). Next, we used the CIBERSORT package to estimate immune cell scores for each sample and computed the correlation between module eigengenes (ME) and immune cell scores. Among the immune-related feature genes, the yellow module exhibited the strongest correlation with immune cells (r = 0.97) (Fig. 2C). We then used volcano maps to show the distribution of differences in gene expression between tumors and normal tissues. A threshold of |log2FC| ≥ 3 and p-value <0.05 was applied to filter genes, resulting in 1026 genes being selected from the GSE58135 dataset. The top 10 up-regulated genes and the top 10 down-regulated genes are displayed(Fig. 2D). Subsequently, we extracted 2831 immune-related genes from the yellow module in the WGCNA analysis and retrieved 23,931 metabolism-related genes from the Genecards database. By taking the intersection of these three gene sets, we ultimately identified 51 immune-metabolic genes (IMGs) (Fig. 2E).

### 3.2 B gene selection through machine learning

**Fig. 3:**
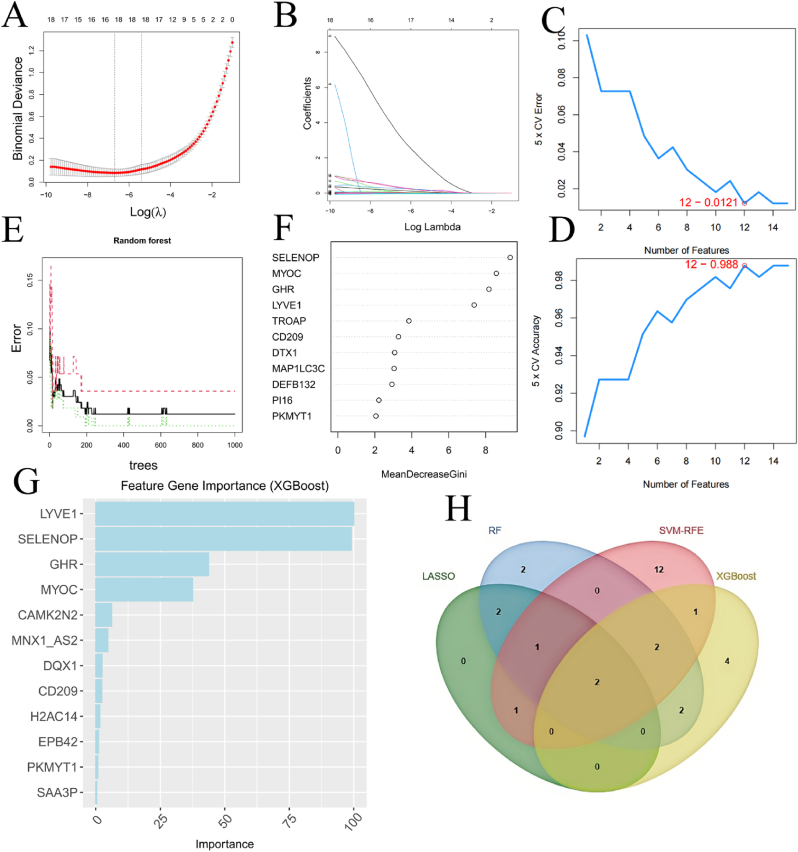
Central genes were screened by machine learning. (A–B) The LASSO algorithm. (C–D) The SVM algorithm. (E–F) The RF algorithm. (G) The XGBoost algorithm. (H) The Venn diagram of the four algorithms.

First, LASSO was applied to filter out 7 genes from the immune-metabolic genes (IMGs)(Fig. 3A-B). Next, the SVM algorithm was used to analyze IMGs, identifying 12 significant genes(Fig. 3C-D). Subsequently, RF analysis was performed, identifying 11 key genes(Fig. 3E-F). The XGBoost algorithm ranked genes based on their importance scores, and we visualized the top 12 feature genes identified by this method(Fig. 3G). Finally, a Venn diagram was used to analyze the intersection of the gene sets selected by LASSO, SVM, RF, and XGBoost. The intersection revealed two genes: SELENOP and PKMYT1(Fig. 3H).

### 3.3 Expression of hub genes and validation with external datasets

**Fig. 4:**
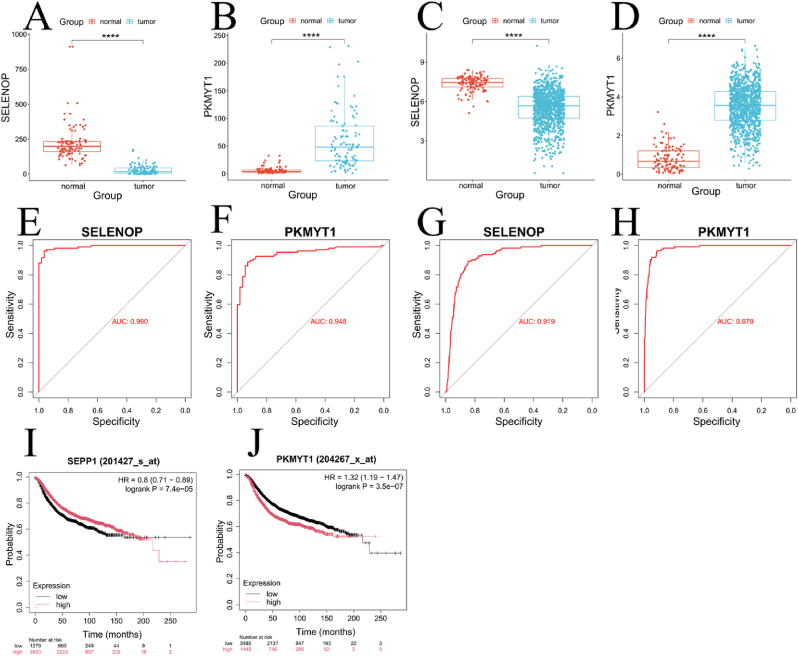
Validation using TCGA Data (A–B) Differential expression of SELENOP and PKMYT1 in the GSE58135 dataset. (C–D) Validation of differential expression for both genes in TCGA cohort. (E–F) Diagnostic potential of SELENOP and PKMYT1 assessed by ROC analysis. (G–H) ROC analysis of SELENOP and PKMYT1 in the TCGA dataset. (I–J) Survival analysis of SELENOP and PKMYT1.

Differential expression analysis was performed to validate the expression of the hub genes (SELENOP and PKMYT1) in external datasets. In the GSE58135 dataset, SELENOP was found to be downregulated, while PKMYT1 was upregulated compared to normal control samples(Fig. 4A-B). Next, we validated these findings using the TCGA breast cancer dataset. The expression patterns of SELENOP and PKMYT1 in TCGA were consistent with those in the GSE58135 dataset, and both genes exhibited statistically significant differences (p < 0.05)(Fig. 4C-D). To assess the diagnostic potential of these genes, ROC analysis was performed. In the GSE58135 dataset, the AUC for SELENOP and PKMYT1 were 0.990 and 0.948, respectively(Fig. 4E-F). In the TCGA dataset, SELENOP and PKMYT1 had AUC values of 0.919 and 0.979, respectively(Fig. 4G-H), suggesting strong diagnostic potential for both genes. Finally, survival analysis was conducted using the Kaplan-Meier Plotter tool (https://kmplot.com/), and the survival curves for SELENOP and PKMYT1 were plotted. The results indicated that high expression of PKMYT1 was associated with poorer prognosis, while high expression of SELENOP was correlated with better survival outcomes(Fig. 4I-J). This further supports the potential of these genes as prognostic markers in breast cancer.

### 3.4 GSEA between high-risk and low-risk groups

**Fig. 5:**
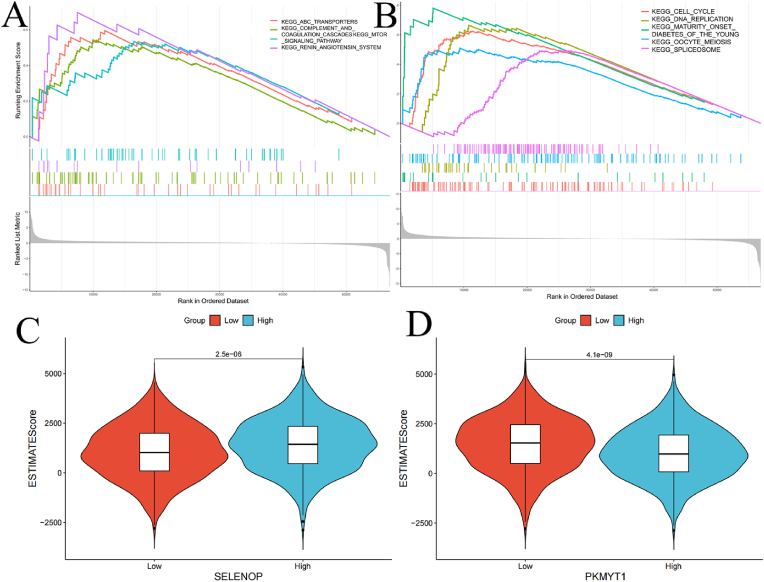
GSEA Analysis and Correlation with ESTIMATE Scores (A–B) GSEA based on the expression levels of SELENOP and PKMYT1. (C–D) Distribution of ESTIMATE scores based on the expression levels of SELENOP and PKMYT1.

To analyze the impact of the high-expression and low-expression groups on cancer progression, we performed Gene Set Enrichment Analysis (GSEA) to identify the most significantly enriched pathways between the two groups. The GSEA results showed that the SELENOP high-expression group was highly enriched in pathways related to ABC transporters, the MTOR signaling pathway, and the coagulation cascade(Fig. 5A). On the other hand, the PKMYT1 high-expression group was highly enriched in pathways related to DNA replication, the cell cycle, spliceosome, and mismatch repair(Fig. 5B). Next, the “ESTIMATE” package was used to compare the ESTIMATE scores between the two groups. As shown in the figure, the SELENOP high-expression group was positively correlated with the ESTIMATE score, while the PKMYT1 high-expression group showed a negative correlation with the ESTIMATE score(Fig. 5C-D).

### 3.5 Immune infiltration analysis

**Fig. 6:**
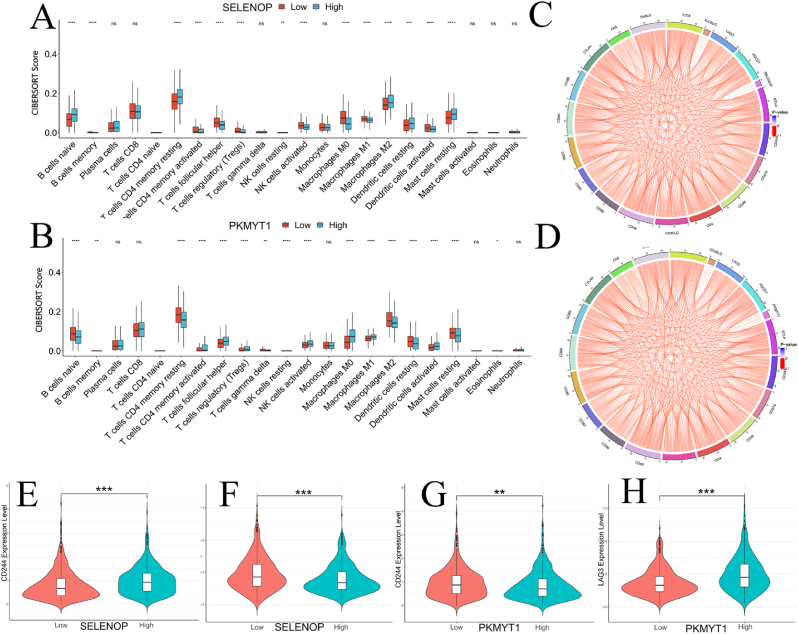
Analysis of Immune Cell Infiltration and its Relationship with SELENOP and PKMYT1 (A–B) Correlation analysis of SELENOP and PKMYT1 expression with 22 immune cell types. (C–D) Relationships between SELENOP/PKMYT1 and immune therapy related molecules visualized by chord diagrams. (E–H) Differential expression of key genes in CD244/LAG-3 high-versus low-expression groups across TCGA and GEO cohorts.

The interaction between tumor development and immune cells is inseparable. To further investigate the relationship between immune cell infiltration and SELENOP and PKMYT1, we used the “CIBERSORT” tool in R to score immune cells in the TCGA dataset. Based on the expression levels of SELENOP and PKMYT1, we divided the samples into high-expression and low-expression groups and analyzed their correlation with various immune cells. The heatmap of 22 immune cell types showed that SELENOP and PKMYT1 genes are correlated with multiple immune cells(Fig. 6A-B). We further analyzed the relationship between immune cells, immune checkpoints, and SELENOP and PKMYT1. The results indicated that FAS, CD28, CD40LG, CD4, CD244, BTLA, CD8A, CD48, CD274, and CD8B were positively correlated with the SELENOP index, while LAG3 and IDO1 were negatively correlated with the SELENOP index(Fig. 6C). On the other hand, LAG3, CD80, PDCD1, IDO1, CTLA4, ICOSLG, and ICOS were positively correlated with the PKMYT1 index, while FAS, CD40LG, TIGIT, CD28, CD244 were negatively correlated with the PKMYT1 index(Fig. 6D). Subsequently, we found that the expressions of CD244 and LAG3 were closely related to the expressions of SELENOP and PKMYT1, and the differences were significant between the high-expression and low-expression groups(Fig. 6E-H). This analysis suggests that both SELENOP and PKMYT1 are involved in the immune microenvironment, influencing the immune cell landscape and immune checkpoint interactions.

### 3.6 Single-cell analysis of BRCA

**Fig. 7:**
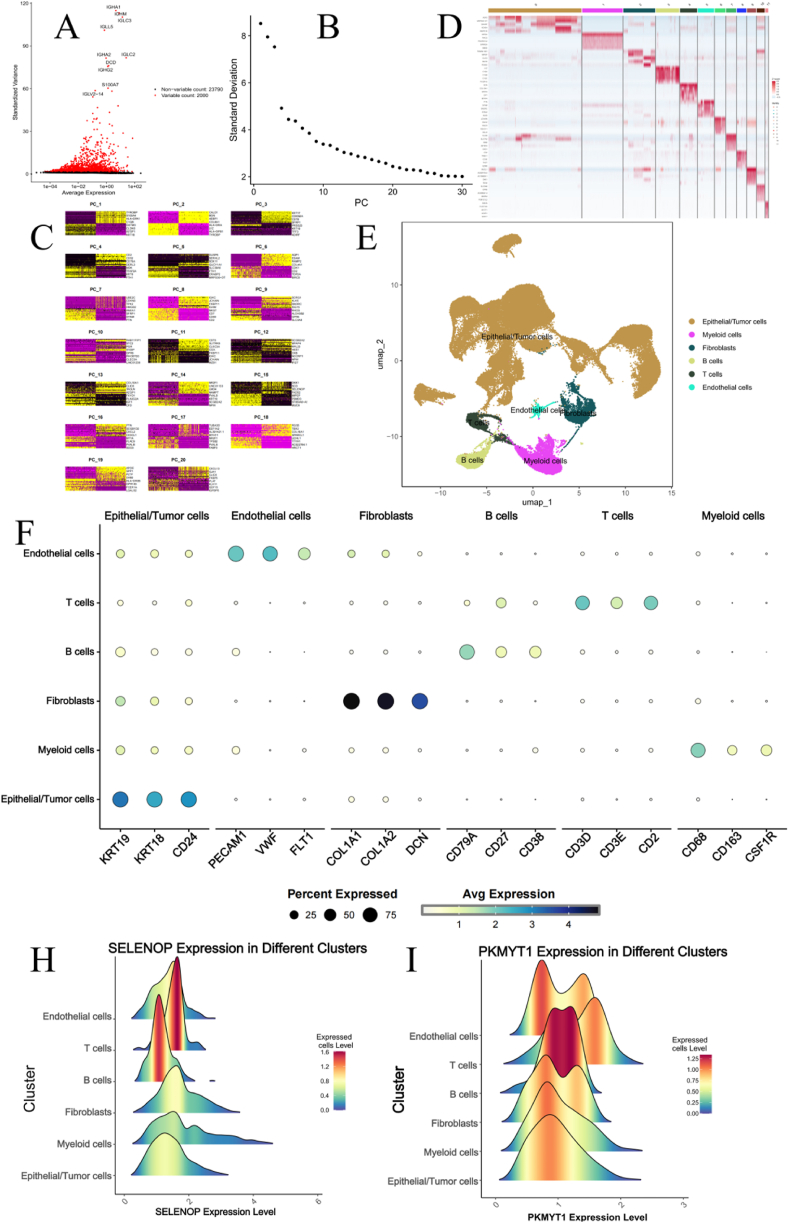
Identification of Six Distinct Annotated Cell Clusters Based on Single-Cell RNA-seq Data (A) Scatter plot of the top 2000 highly variable genes. (B) ElbowPlot of 28 samples. (C) PC Heatmap of the top 20 PCs. (D) The five marker genes with the highest average logFC in each subgroup. (E) Twelve cell clusters were identified as six types of cells. (F) Based on the composition of marker genes, the 12 cell clusters were identified as six distinct cell types. (H–I) Expression levels of SELENOP and PKMYT1 in each cell cluster.

We selected 28 single-cell RNA sequencing samples from the GSE161529 dataset. First, we filtered out low-quality cells based on quality control standards (with a mitochondrial gene proportion of 15 %), ultimately obtaining 87,597 high-quality cells for subsequent analysis. Then, we identified 2000 highly variable genes for downstream dimensionality reduction(Fig. 7A). In addition, we selected 9 principal components (PCS) based on ElbowPlot and PC Heatmap, and divided the samples into 12 cell clusters(Fig. 7B-C). Use the clustering heat map to display the top 5 marker genes of each subgroup(Fig. 7D). Using the “SingleR” package, we annotated the cell clusters based on marker genes and identified six cell types: epithelial cells, endothelial cells, fibroblasts, myeloid cells, T cells, and B cells(Fig. 7E). The expression of key marker genes for each cell type was visualized using a bubble plot(Fig. 7F). SELENOP was primarily expressed in T cells and B cells, while the PKMYT1 gene was mainly expressed in endothelial cells, T cells, and B cells(Fig. 7H-I).

### 3.7 Immunohistochemical analysis

**Fig. 8:**
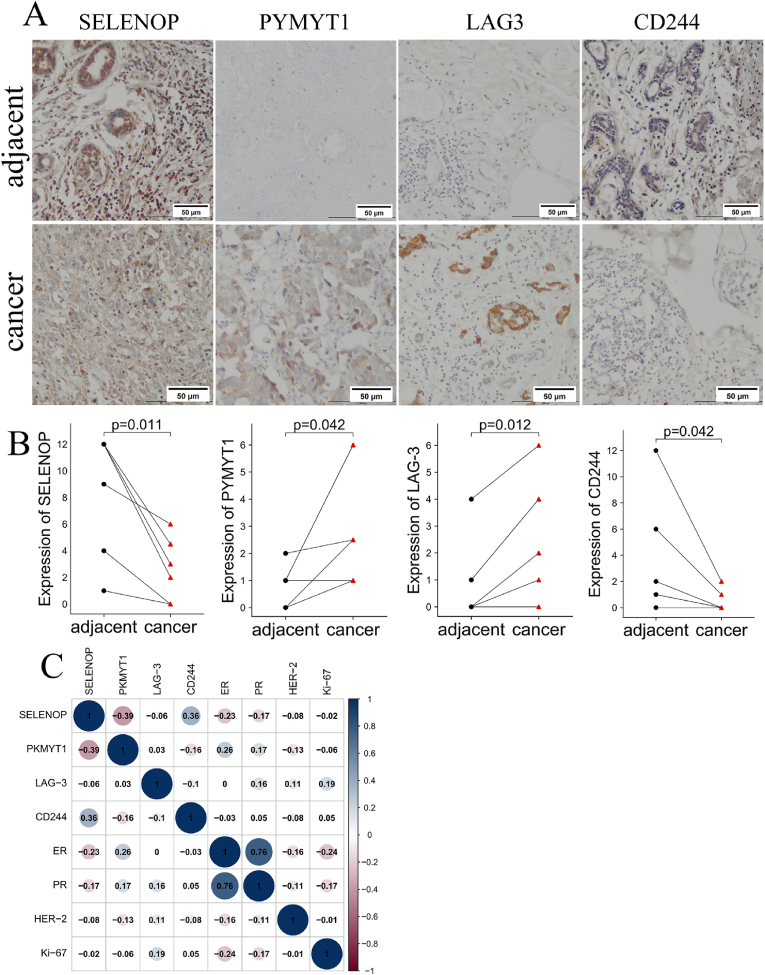
IHC Validation (A) Representative IHC images of SELENOP, PKMYT1, LAG-3, and CD244. (B) Analysis of expression differences of SELENOP, PKMYT1, LAG-3 and CD244 in adjacent tissues and cancer. (C) Correlation analysis between SELENOP, PKMYT1, LAG-3, CD244, ER, PR, HER-2 and Ki-67.

We collected breast cancer tissue samples from 60 volunteers and performed IHC experiments to validate the expression of SELENOP, PKMYT1 and immune checkpoints in BRCA tissue. Considering the expression distribution characteristics of SELENOP and PKMYT1, we chose the median value to divide the high and low expression groups to ensure group balance and statistical power. We found significant differences in the expression of SELENOP, PKMYT1, LAG3 and CD244 between the cancer tissues and adjacent cancer tissues. SELENOP and CD244 were highly expressed in the adjacent cancer tissues, while PKMYT1 and LAG3 were highly expressed in the cancer tissues(Fig. 8A-B). Subsequently, a correlation analysis of these genes was performed. The results showed that SELENOP was negatively correlated with PKMYT1, LAG-3, ER, PR, HER-2 and Ki-67, while it was positively correlated with CD244. The results showed that PKMYT1 was negatively correlated with SELENOP, CD244, HER-2 and Ki-67, while it was positively correlated with LAG-3,ER and PR(Fig. 8C). The expression of SELENOP was not statistically significant concerning patient age (p > 0.05); however, differences in histological grade, lymph node metastasis, LAG-3, CD244, estrogen receptors, progesterone receptors, and Her-2 expression were statistically significant (p < 0.05) (Table 1). The expression of PKMYT1 was not statistically significant concerning patient age, and lymph node metastasis (p > 0.05); however, differences in histological grade, LAG-3, CD244, estrogen receptors, progesterone receptors, and Her-2 expression were statistically significant (p < 0.05) (Table 1).

**Table 1:** Correlation between the expression of SELENOP and PKMYT1 and clinico-pathological parameters.

Variable	n	SELENOP (n)		*P* value	PKMYT1 (n)		*P* value
		High expression	Low expression		High expression	Low expression	
Age (years)							
≤50	22	12	10	0.762	10	12	0.348
>50	38	18	20		20	18	
Grade							
Ⅰ∼Ⅱ	47	29	18	0.033	18	29	<0.001
Ⅲ	13	1	12		12	1	
Lymph node							
0∼3	42	24	18	0.018	20	22	0.207
>3	18	6	12		10	8	
LAG-3							
0∼1	35	23	12	0.039	11	24	0.007
>1	25	7	18		19	6	
CD244							
0∼1.5	26	5	21	<0.001	19	8	0.017
>1.5	34	25	9		11	23	
Estrogen receptor							
	22	15	7	0.001	12	10	0.036
+	38	15	23		18	20	
Progesterone							
	34	20	14	0.002	16	18	<0.001
+	26	10	16		14	12	
Her-2							
	13	9	4	0.034	2	11	0.045
+	47	21	26		28	19	

## 4 Discussion

The occurrence of breast cancer is the result of the combined effects of various factors, including genetics, environment, hormones, and lifestyle, and the specific molecular mechanisms are still under investigation [20]. Studies have shown that the progression of breast cancer is closely related to immune system responses and metabolic regulation, both of which play important roles in tumor growth and metastasis [21]. Several studies using WGCNA have revealed key genes and their potential molecular mechanisms in breast cancer, providing new insights into the pathophysiological processes of the disease [22]. After the preliminary identification of differentially expressed genes (DEGs), we combined WGCNA and machine learning techniques to further screen potential biomarkers. We identified two immune-metabolic hub genes, SELENOP and PKMYT1, and validated them using the TCGA dataset. We performed time-dependent ROC curve analysis to evaluate the diagnostic value of these hub genes.

Selenoprotein P (SELENOP) is a protein that contains multiple selenium-cysteine residues and is primarily responsible for selenium transport in the body. It also plays a crucial role in antioxidant defense [23]. Numerous experimental studies have shown that selenium can inhibit the growth and proliferation of cancer cells through various mechanisms, including the regulation of oxidative stress and the modulation of signaling pathways [24,25]. In breast cancer research, selenium has been found to enhance the anticancer effect of mouse breast cancer 4T1 cells by regulating the signaling pathways of androgen receptor (AR), insulin-like growth factor-1 receptor (IGF-1R), epidermal growth factor receptor (EGFR), and calcium-binding protein TROP2 [26]. Additionally, another study indicated that methyl selenite significantly inhibits breast cancer growth by suppressing the JAK2/STAT3 signaling pathway [27]. We found that in the high SELENOP expression group, the PI3K/AKT/mTOR signaling pathway was upregulated. This may be consistent with SELENOP's role in protecting cells from oxidative damage by neutralizing free radicals, thus reducing cancer risk.

PKMYT1 is a membrane-associated protein kinase that regulates the progression of the cell cycle mainly by phosphorylating specific sites on cyclin-dependent kinase 1 (CDK1) [28]. It is generally believed that PKMYT1 inhibits CDK1 activity by phosphorylating Thr14 and Tyr15 of CDK1, preventing cells from entering mitosis (M phase) [29]. A proteogenomic analysis based on patient-derived xenografts (PDX) from 22 ER + breast cancer patients suggested that PKMYT1 is a WEE1 homolog [30]. WEE1 can phosphorylate Tyr15 of CDK1 and is involved in regulating the activity of CDK1 and CDK2 [29]. In tumor cells, the mutation rate of the tumor suppressor gene p53 is relatively high, with mutations found in over 50 % of tumor cells [31]. Tumor cells with p53 dysfunction exhibit inactivation of the G1/S checkpoint [32], making DNA repair largely dependent on the G2/M checkpoint. At this point, inhibiting WEE1 further inactivates the G2/M checkpoint [33], driving cells to prematurely enter mitosis, resulting in mitotic catastrophe and causing a synthetic lethality effect (SL) [34]. This finding is consistent with our functional enrichment analysis, which showed that PKMYT1 plays a key role in cell cycle regulation and DNA damage repair. In this study, we found that PKMYT1 expression was significantly elevated in breast cancer tissues and demonstrated high diagnostic accuracy for breast cancer, suggesting its potential diagnostic value.

Two key interconnected mechanisms driving cancer progression are immune escape and metabolic reprogramming. Our findings demonstrate distinct cellular distributions for SELENOP and PKMYT1, with SELENOP predominantly expressed in immune cells and PKMYT1 widely expressed in endothelial cells, suggesting their involvement in microenvironment regulation through different cellular compartments. High SELENOP expression shows significant positive correlations with CD8^+^ T cell infiltration and immune activating molecules and significant negative correlations with immunosuppressive molecules. These observations provide novel insights, indicating that dysregulation of selenium metabolism, as reflected by SELENOP expression, may contribute to immune escape by modulating immune cell activity. Notably, high PKMYT1 expression is significantly enriched in poorly differentiated tumors and shows a positive correlation with Estrogen Receptor and Progesterone Receptor status. This pattern suggests PKMYT1 holds potential as a clinical biomarker relevant to tumor grading and molecular subtyping.

This study has certain limitations. Firstly, although RNAseq analysis based on public databases combines immunohistochemical validation of independent cocohort (n = 60), the statistical power of subgroup analysis may still be limited. Secondly, the mechanisms of SELENOP and PKMYT1 in the occurrence and development of breast cancer remain unclear, which will be the focus of subsequent experimental research.

## 5 Conclusion

This study establishes SELENOP and PKMYT1 as key metabolic-immune regulators reprogramming the breast cancer microenvironment, serving as diagnostic biomarkers and therapeutic targets.

## CRediT authorship contribution statement

**Guohui Tang:** Writing – original draft, Methodology, Conceptualization. **Zheng Zhang:** Investigation, Formal analysis. **Bo Pang:** Software, Formal analysis. **Ruonan Li:** Resources, Data curation. **Yuting Liu:** Resources, Data curation. **Haotian Cai:** Resources, Data curation. **Wenrui Wang:** Visualization, Supervision. **Changjie Chen:** Visualization, Supervision. **Yurong Ou:** Visualization, Supervision. **Qingling Yang:** Writing – review & editing, Funding acquisition.

## Ethical approval

This study was approved by the Ethics Committee of Bengbu Medical University (Reference number: 2023-236). The patient provided their written informed consent to participate in this study.

## Ethics statement

The study was approved by the Ethics Committee of Bengbu Medical College.

## Funding

This study was supported by the University Synergy Innovation Program of Anhui Province (GXXT-2022-064), excellent Scientific Research and Innovation Team of Anhui Universities (2024AH010021), the Major Program of Anhui Educational Committee (No.: KJ2019ZD28), the Program for graduate's research of Bengbu Medical College (Byycx22042, Byycx23013).

## Declaration of Competing interest

The authors declare no conflict of interest.

## Data Availability

The information of the clinical samples used in this article is provided in the attachment.

## References

[1] Łukasiewicz S., Czeczelewski M., Forma A., Baj J., Sitarz R., Stanisławek A. (2021 Aug 25). Breast cancer-epidemiology, risk factors, classification, prognostic markers, and current treatment Strategies-An updated review. Cancers (Basel).

[2] Zhang D., Xu X., Ye Q. (2021 Apr). Metabolism and immunity in breast cancer. Front. Med..

[3] Oh D.Y., Fong L. (2021 Dec 14). Cytotoxic CD4+ T cells in cancer: expanding the immune effector toolbox. Immunity.

[4] Bates J.P., Derakhshandeh R., Jones L., Webb T.J. (2018 May 11). Mechanisms of immune evasion in breast cancer. BMC Cancer.

[5] Kao K.C., Vilbois S., Tsai C.H., Ho P.C. (2022 Nov). Metabolic communication in the tumour-immune microenvironment. Nat. Cell Biol..

[6] Wang B., Pei J., Xu S., Liu J., Yu J. (2024 Mar 8). A glutamine tug-of-war between cancer and immune cells: recent advances in unraveling the ongoing battle. J. Exp. Clin. Cancer Res..

[7] Wang Z., Jiang Q., Dong C. (2020 Feb 15). Metabolic reprogramming in triple-negative breast cancer. Cancer Biol Med.

[8] Schomburg L. (2022 Oct). Selenoprotein P - Selenium transport protein, enzyme and biomarker of selenium status. Free Radic. Biol. Med..

[9] Cui X., Liu S., Song H., Xu J., Sun Y. (2025 Apr 9). Single-cell and spatial transcriptomic analyses revealing tumor microenvironment remodeling after neoadjuvant chemoimmunotherapy in non-small cell lung cancer. Mol. Cancer.

[10] Oliveira M.F., Romero J.P., Chung M., Williams S.R., Gottscho A.D., Gupta A., Pilipauskas S.E., Mohabbat S., Raman N., Sukovich D.J., Patterson D.M., Visium H.D., Taylor S.E.B., Development Team (2025 Jun). High-definition spatial transcriptomic profiling of immune cell populations in colorectal cancer. Nat. Genet..

[11] Tomović Pavlović K., Kocić G., Šmelcerović A. (2024 Mar 1). Myt1 kinase inhibitors - insight into structural features, offering potential frameworks. Chem. Biol. Interact..

[12] Farhadian M., Rafat S.A., Panahi B., Mayack C. (2021 Jan 27). Weighted gene co-expression network analysis identifies modules and functionally enriched pathways in the lactation process. Sci. Rep..

[13] Antonelli J., Parmigiani G., Dominici F. (2019 Sep). High-dimensional confounding adjustment using continuous spike and slab priors. Bayesian Anal.

[14] Pourakbar N., Motamedi A., Pashapour M., Sharifi M.E., Sharabiani S.S., Fazlollahi A., Abdollahi H., Rahmim A., Rezaei S. (2025 Jun 5). Effectiveness of artificial intelligence models in predicting lung cancer recurrence: a gene biomarker-driven review. Cancers (Basel).

[15] Taha H.A., Zeilani R.S., Haddad R.H., Abdalrahim M.S. (2025 Jul 7). Artificial intelligence and machine learning techniques for predicting neuropathic pain in patients with cancer: a systematic review. Digit Health.

[16] Kiruba B., Narayan P.S.A., Raj B., Raj S.R., Mathew S.G., Lulu S.S., Sundararajan V. (2025 Jun 5). Intervention of machine learning in bladder cancer research using multi-omics datasets: systematic review on biomarker identification. Discov. Oncol..

[17] Győrffy B. (2024 Apr 9). Integrated analysis of public datasets for the discovery and validation of survival-associated genes in solid tumors. Innovation.

[18] Dakal T.C., George N., Xu C., Suravajhala P., Kumar A. (2024 Apr 23). Predictive and prognostic relevance of tumor-infiltrating immune cells: tailoring personalized treatments against different cancer types. Cancers (Basel).

[19] Kim S.Y., Volsky D.J. (2005 Jun 8). PAGE: parametric analysis of gene set enrichment. BMC Bioinf..

[20] Feng Y., Spezia M., Huang S., Yuan C., Zeng Z., Zhang L., Ji X., Liu W., Huang B., Luo W., Liu B., Lei Y., Du S., Vuppalapati A., Luu H.H., Haydon R.C., He T.C., Ren G. (2018 May 12). Breast cancer development and progression: risk factors, cancer stem cells, signaling pathways, genomics, and molecular pathogenesis. Genes Dis.

[21] Zhou Y., Wang H., Luo Y., Tuo B., Liu X., Li T. (2023 Mar). Effect of metabolism on the immune microenvironment of breast cancer. Biochim. Biophys. Acta Rev. Canc.

[22] Xu L., Wang S., Zhang D., Wu Y., Shan J., Zhu H., Wang C., Wang Q. (2023 Dec). Machine learning- and WGCNA-Mediated double analysis based on genes associated with disulfidptosis, cuproptosis and ferroptosis for the construction and validation of the prognostic model for breast cancer. J. Cancer Res. Clin. Oncol..

[23] Saito Y. (2021 May 28). Selenium transport mechanism via selenoprotein P-Its physiological role and related diseases. Front. Nutr..

[24] Vinceti M., Filippini T., Del Giovane C., Dennert G., Zwahlen M., Brinkman M., Zeegers M.P., Horneber M., D'Amico R., Crespi C.M. (2018 Jan 29). Selenium for preventing cancer. Cochrane Database Syst. Rev..

[25] Rataan A.O., Geary S.M., Zakharia Y., Rustum Y.M., Salem A.K. (2022 Feb 17). Potential role of selenium in the treatment of cancer and viral infections. Int. J. Mol. Sci..

[26] Guo C.H., Wang S.Y., Chung C.H., Shih M.Y., Li W.C., Chen P.C., Lee S.Y., Hsia S. (2023 Oct). Selenium modulates AR/IGF-1R/EGFR and TROP2 signaling pathways and improves anticancer efficacy in murine mammary carcinoma 4T1. J. Nutr. Biochem..

[27] Qiu C., Zhang T., Zhu X., Qiu J., Jiang K., Zhao G., Wu H., Deng G. (2019 Jun). Methylseleninic acid suppresses breast cancer growth via the JAK2/STAT3 pathway. Reprod. Sci..

[28] Yang M., Xiang H., Luo G. (2024 Oct 9). Targeting protein kinase, membrane-associated tyrosine/threonine 1 (PKMYT1) for precision cancer therapy: from discovery to clinical trial. J. Med. Chem..

[29] Szychowski J., Papp R., Dietrich E., Liu B., Vallée F., Leclaire M.E., Fourtounis J., Martino G., Perryman A.L., Pau V., Yin S.Y., Mader P., Roulston A., Truchon J.F., Marshall C.G., Diallo M., Duffy N.M., Stocco R., Godbout C., Bonneau-Fortin A., Kryczka R., Bhaskaran V., Mao D., Orlicky S., Beaulieu P., Turcotte P., Kurinov I., Sicheri F., Mamane Y., Gallant M., Black W.C. (2022 Aug 11). Discovery of an orally bioavailable and selective PKMYT1 inhibitor, RP-6306. J. Med. Chem..

[30] Chen A., Kim B.J., Mitra A., Vollert C.T., Lei J.T., Fandino D., Anurag M., Holt M.V., Gou X., Pilcher J.B., Goetz M.P., Northfelt D.W., Hilsenbeck S.G., Marshall C.G., Hyer M.L., Papp R., Yin S.Y., De Angelis C., Schiff R., Fuqua S.A.W., Ma C.X., Foulds C.E., Ellis M.J. (2024 Oct 1). PKMYT1 is a marker of treatment response and a therapeutic target for CDK4/6 inhibitor-resistance in ER+ breast cancer. Mol. Cancer Therapeut..

[31] Hernández Borrero L.J., El-Deiry W.S. (2021 Aug). Tumor suppressor p53: biology, signaling pathways, and therapeutic targeting. Biochim. Biophys. Acta Rev. Canc.

[32] Benedict B., van Harn T., Dekker M., Hermsen S., Kucukosmanoglu A., Pieters W., Delzenne-Goette E., Dorsman J.C., Petermann E., Foijer F., Te Riele H. (2018 Oct 16). Loss of p53 suppresses replication-stress-induced DNA breakage in G1/S checkpoint deficient cells. eLife.

[33] Schmidt M., Rohe A., Platzer C., Najjar A., Erdmann F., Sippl W. (2017 Nov 23). Regulation of G2/M transition by inhibition of WEE1 and PKMYT1 kinases. Molecules.

[34] Kciuk M., Gielecińska A., Mujwar S., Mojzych M., Kontek R. (2022 Mar 24). Cyclin-dependent kinase synthetic lethality partners in DNA damage response. Int. J. Mol. Sci..

